# Trapped tidal currents generate freely propagating internal waves at the Arctic continental slope

**DOI:** 10.1038/s41598-023-41870-3

**Published:** 2023-09-08

**Authors:** Till M. Baumann, Ilker Fer

**Affiliations:** 1 Geophysical Institute, University of Bergen and Bjerknes Centre for Climate Research, Bergen, Norway; 2 Present Address: Institute for Marine Research, Bergen, Norway

**Keywords:** Ocean sciences, Physical oceanography

## Abstract

Energetic tidal currents in the Arctic play an important role in local mixing processes, but they are primarily confined to the shelves and continental slopes due to topographic trapping north of their critical latitude. Recent studies employing idealized models have suggested that the emergence of higher harmonic tidal waves along these slopes could serve as a conduit for tidal energy transmission into the Arctic Basin. Here we provide observational support from an analysis of yearlong observations from three densely-instrumented oceanographic moorings spanning 30 km across the continental slope north of Svalbard ($\sim$81.3$^{\circ }$N). Full-depth current records show strong barotropic diurnal tidal currents, dominated by the K$_1$ constituent. These sub-inertial currents vary sub-seasonally and are strongest at the 700-m isobath due to the topographic trapping. Coinciding with the diurnal tide peak in summer 2019, we observe strong baroclinic semidiurnal currents exceeding 10 cm s$^{-1}$ between 500 m and 1000 m depth about 10 km further offshore at the outer mooring. In this semidiurnal band, we identify super-inertial K$_2$ waves, and present evidence that their frequency, timing, polarization, propagation direction and depths are consistent with the generation as higher harmonics of the topographically trapped K$_1$ tide at the continental slope.

## Introduction

The ocean internal wave field, its associated energy dissipation and subsequent mixing are a key control of global ocean circulation and stratification^1,2^. Internal waves originate primarily from direct forcing of the stratified ocean by wind variations at the surface or by tidal flow over topography. Abundant barotropic (i.e. water-depth independent) tidal currents interact with topography and stratification, thus creating internal waves at tidal frequencies.

Linear internal waves can transfer their energy vertically through the vertical component of their group velocity. However, to propagate freely, waves with frequency $\omega$ must satisfy $f\le \omega \le N$, in the absence of background vorticity. Here *f* is the local Coriolis parameter and *N* is the Brunt-Väisälä frequency, a measure of vertical stratification in the ocean. Lower frequency, sub-inertial (i.e. $\omega <f$) waves can propagate along topographic wave guides. As $f=2\,\Omega \,$sin$(\phi )$ changes with latitude ($\phi$), where $\Omega =7.2921159 \times 10^{-5}$ rad s$^{-1}$ is the Earth’s angular velocity, the critical latitude beyond which the waves cannot freely propagate is 30$^{\circ }$N and 27.37$^{\circ }$N for the dominant diurnal tidal constituents K$_1$ and O$_1$, and 74.46 $^{\circ }$N and 85.7$^{\circ }$N, for the dominant semidiurnal tidal constituents M$_2$ and S$_2$, respectively. The region studied here, the continental slope north of Svalbard ($\sim$81.3$^{\circ }$N), and indeed most of the deep Arctic Ocean are consequently north of the critical latitude of all dominant tidal constituents, except for the relatively weak S$_2$.

Under favorable conditions, e.g. when the cross-isobath component of the tidal current is energetic enough to induce vertical displacements of density surfaces, sub-inertial barotropic tides can energize waves that will be “trapped” along topography. Trapped diurnal tides are regarded as a major source of energy for mixing around steep topography such as sea mounts throughout much of the world ocean (poleward of 30$^{\circ }$)^3,4^. In the Arctic Ocean, the M$_2$ tide does also contribute to a substantial energy transfer from barotropic tides to topographically trapped waves^3,5^. This trapped energy is thought to contribute to water mass transformation within the Arctic Circumpolar Boundary Current^6,7^, but its effects are mainly localized at the continental slope.

Yet, there exist mechanisms for the energy of topographically trapped tidal waves to travel away from the slope. For one, strong tidal currents flowing over a shelf break may create lee waves which, upon slackening of the tidal flow, can propagate as highly energetic nonlinear internal waves. These waves have been studied extensively in recent years in the Arctic due to their strong potential to generate mixing^8–13^. Recent studies suggest these waves are rather abundant along the (eastern) Arctic continental slope^11,12^.

Energy can also be extracted from trapped tides in the form of higher harmonic internal waves, whose frequencies are integer multiples of the originating (tidal) wave’s frequency. When the higher harmonic frequencies become super-inertial (i.e. $\omega >f$), there arises the possibility for free propagation, including in the offshore direction. 3-D numerical experiments indicate, however, that rather than an abrupt transition, there is a gradual shift from a mostly trapped internal wave field to increasingly free 3-D propagation as frequencies increase from sub- to super-inertial^14^. The principle of harmonic generation has been shown in theory^15–17^, model simulations^18^, and tank experiments^19,20^. Although questions remain about the precise generating mechanism, studies suggest that the most efficient generation occurs where the topographic slope angle closely matches the aspect ratio (or angle) of the internal wave^20,21^. The energy of the harmonic waves decreases as their frequency increases, thus favoring low numbered higher harmonics (e.g. 2, 3). However, north of the critical latitude of the fundamental wave, the amplitude of any harmonics is thought to be small^22^. Recent idealized simulations based on the Yermak Plateau continental slope and realistic diurnal tidal forcing show the generation of super-inertial K$_2$ (i.e. 2K$_1$) waves^21^. Oscillations at higher harmonic frequency of the dominant tidal waves have also been observed in moored records around the Yermak Plateau region^23,24^. Using 3 years of velocity records at the top of the Yermak Plateau, Artana et al.^24^ observe strong semidiurnal shear despite dominating diurnal tidal velocities. They propose that the enhanced shear around the semidiurnal band could be attributed to the generation of internal waves at 2K$_1$ frequency, forced by the flow of diurnal tides over critical slopes around the plateau. Similarly, on the southern flanks of the Yermak Plateau, using year-long moored observations supplemented by 2D numerical simulations, Wang et al.^23^ note the generation of higher harmonics of diurnal tidal forcing in their analysis of internal wave energy properties. Despite assigning a large proportion of energy radiation to these higher harmonic waves, they estimate that most of the energy is dissipated locally. Here we present observational evidence suggesting the generation site and possible propagation trajectories of substantial second harmonic (and thus super-inertial) internal waves, likely originating from trapped K$_1$ tidal waves at the continental slope north of Svalbard.

**Figure 1:**
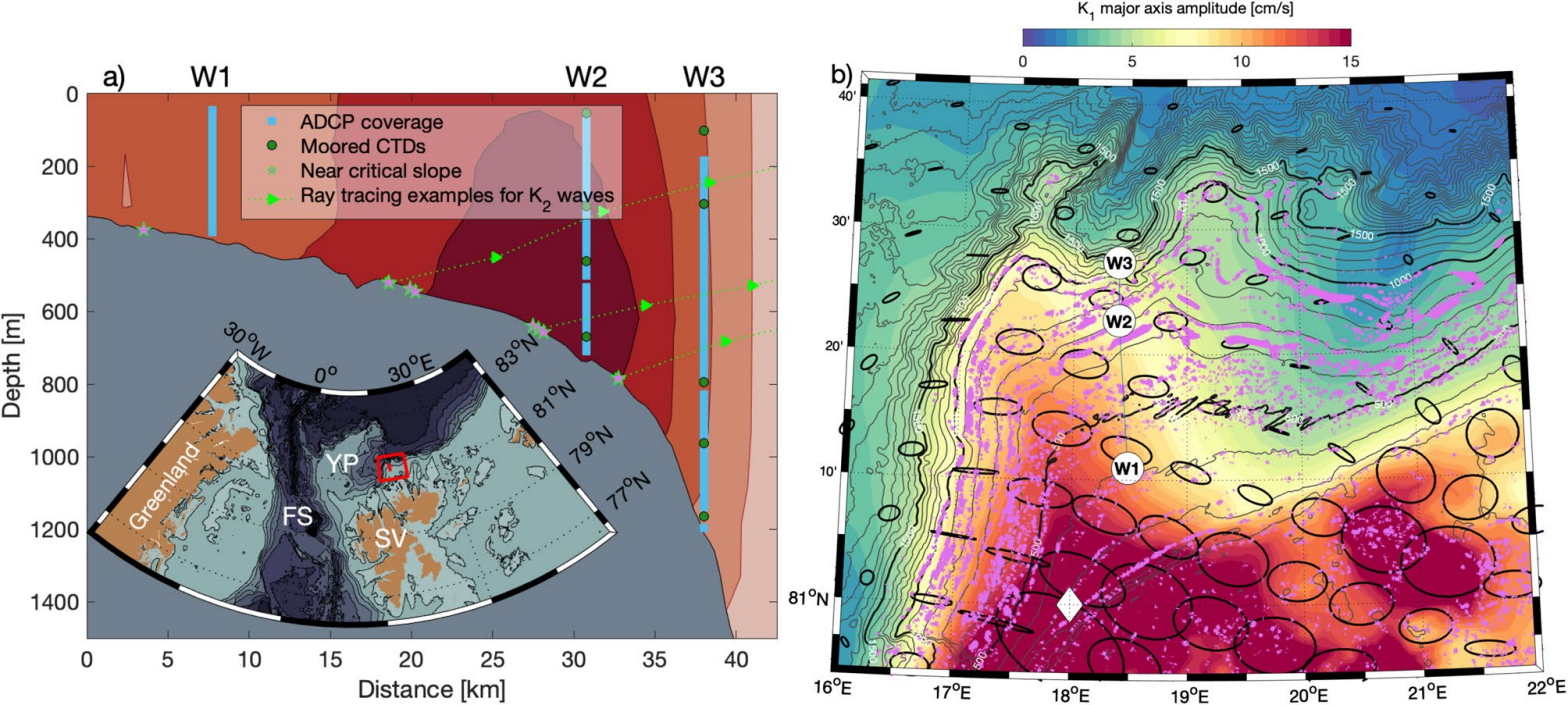
(**a**) Location of the W-moorings across the continental slope and their instrumentation in terms of ADCP coverage (blue lines) and CTDs (green dots). Shading indicates the shape and amplitude (darker with increased amplitude) of the cross-slope velocity of the trapped diurnal (K$_1$) tidal wave obtained from an idealized model solving the eigenvalue problem for trapped waves^25^. Purple stars indicate slopes whose criticality parameter $\alpha$ (Eq. 1) is within 10% of critical with respect to K$_2$, the second harmonic of K$_1$. Dotted lines are examples of trajectories of K$_2$ generated at the slope. Insert: regional map around the study area (red), topography is shaded. Labels YP, SV and FS indicate the locations of the Yermak Plateau, Svalbard and Fram Strait, respectively. (**b**) Detailed map of the surroundings of the moorings (are marked in red in the insert in (**a**)). Color shading shows major axis amplitudes of K$_1$ at the continental slope North of Svalbard obtained from the Arc2km Arctic tide model^26^, with examples of tidal ellipse shapes drawn in black. Mooring locations are marked and labelled W1 to W3. The white diamond indicates the site of previous observations of tidally generated non-linear internal waves^12^. Purple shading indicates near-critical slope angles as in (**a**).

## Data and methods

**Figure 2:**
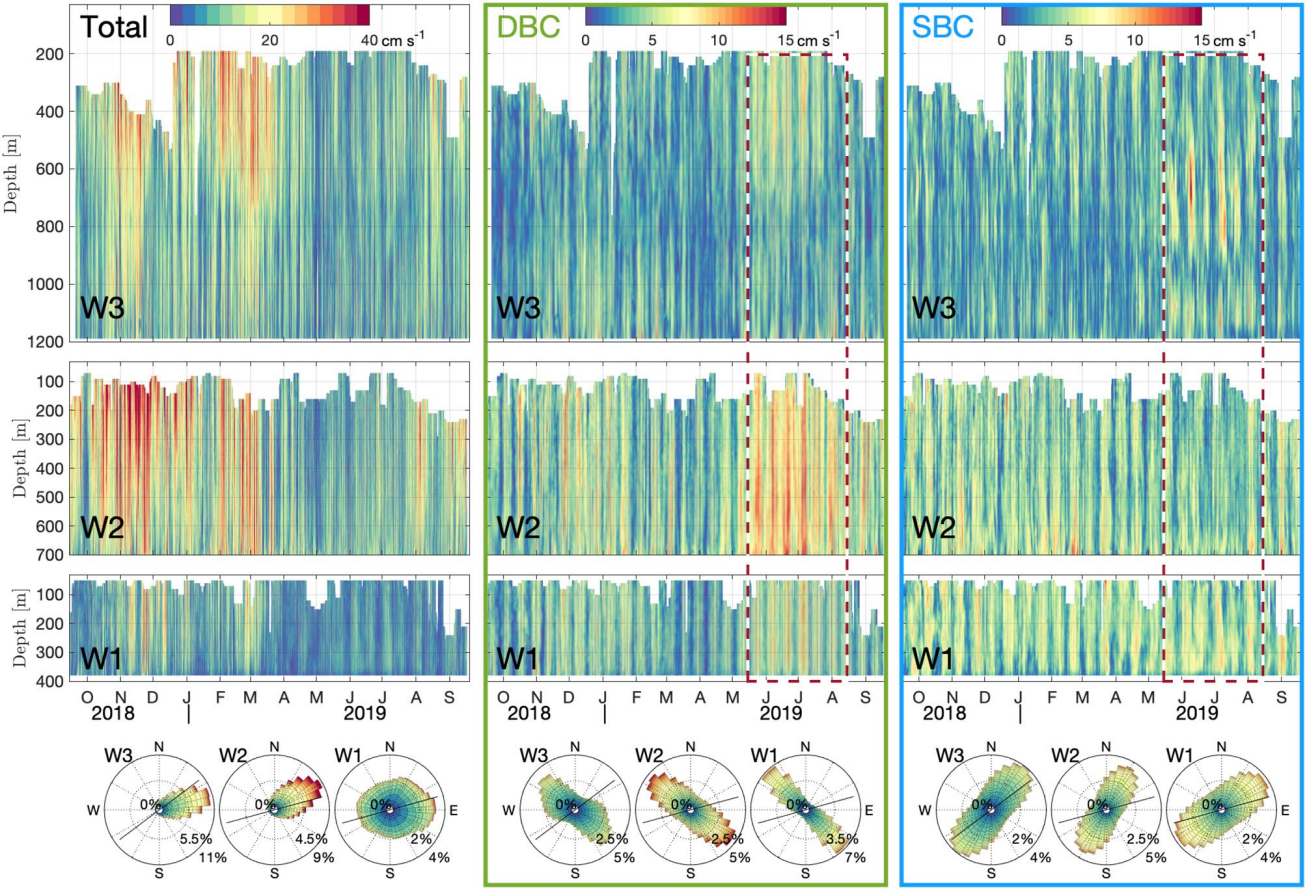
Time-depth plots of total currents (left), diurnal band currents (DBCs, middle) and semidiurnal band currents (SBCs, right). Colors indicate current speeds. Dashed red lines mark the time interval of strongest DBC. Roses show the direction of each current measurement in 10$^{\circ }$ bins, colored according to their speed; the distance from the center is proportional to the occurrence of the specific direction bin expressed in percent. Black lines indicate the isobath orientation at the mooring location, estimated from the IBCAO version 4 topography.

An array of three moorings was deployed across the continental slope north of Svalbard between September 2018 and September 2019. The three moorings (W1, W2 and W3) were located at approximately 400 m, 700 m and 1200 m isobaths. The array was designed to resolve the Atlantic Water (AW) structure and its variability and was instrumented to obtain near full-depth velocity observations beneath the surface mixed layer (Fig. 1a). While at W1 a single upward looking 75kHz RDI ADCP was sufficient to monitor the whole water column, W2 and W3 were equipped with two ADCPs each. At W2, a 75 kHz and a 150kHz RDI ADCP were deployed in the same frame at 525 m depth, with transducers pointing upward and downward, respectively. At W3 a similar setup was used to moor a pair of Nortek ADCPs pointing upward (55 kHz) and downward (150 kHz) at 800 m depth. The ADCPs recorded data at a sampling interval of 1 hour, except for the 150 kHz ADCP at W2, which operated at 20-minute intervals. The instrumentation and processing is described in detail in a report^27^ and the data set is freely accessible^28^. The observed velocities are dominated by the boundary current, transporting AW into the Arctic Ocean. The boundary current variability has been discussed before^29^ and exhibits a substantial seasonal cycle with peak velocities in winter and minima in summer (Fig. 2).

During the mooring deployment cruise, 67 ship-based Conductivity–Temperature–Depth (CTD) profiles were conducted in the area^30^. We use this hydrographic data for the calculation of slope criticality in the region.

Current variability in different bands is analyzed using a 5th order Butterworth band-pass filter over periods including only the diurnal band currents (DBC, 19-30h), semidiurnal band currents (SBC, 10-15h) and the S$_2$+K$_2$ frequency band (11.8-12.2h, these two constituents are closely spaced in frequency and cannot be separated using band-pass filters).

**Figure 3:**
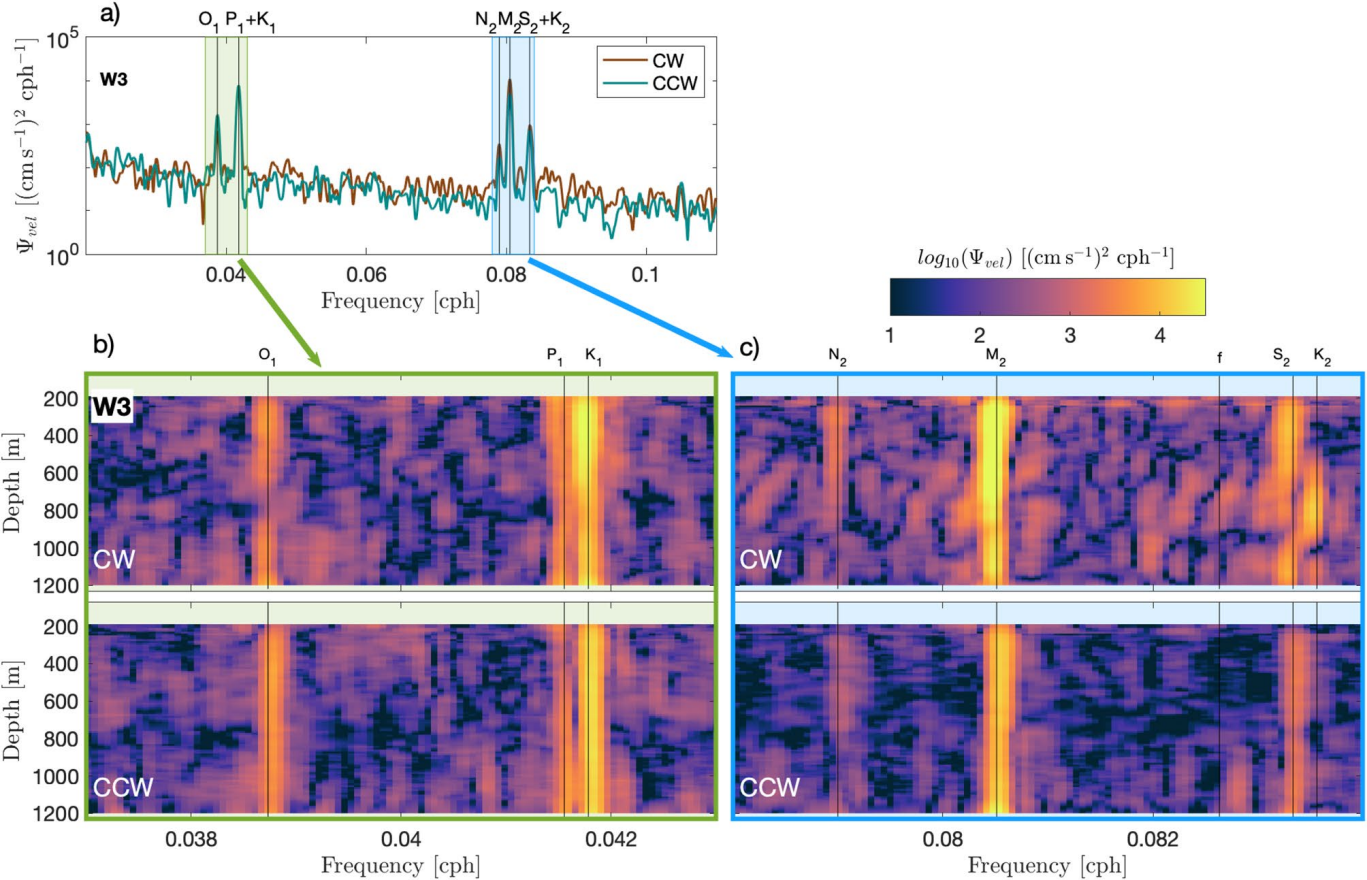
Rotary spectra of year-long velocity time series at W3 for vertically averaged currents (**a**) and at each depth level (**b**, **c**). The panels in (**b**) are focused on the diurnal band, while the panels in (**c**) display the semidiurnal band. Major tidal constituents as well as the inertial frequency *f* are marked with vertical lines and labels at the top.

Spectral analysis is conducted to obtain two-sided power spectral density estimates, accounting for both clockwise (CW) and counter-clockwise (CCW) rotary components. For spectra of depth-averaged currents (such as in Fig. 3a), the time series is partitioned into 50% overlapping segments that are Hanning windowed, utilizing Welch’s method to enhance spectral amplitude accuracy. When the aim is to distinguish between constituents closely spaced in frequency (such as P$_1$ and K$_1$ as well as S$_2$ and K$_2$), we use the complete (un-segmented) year-long record to achieve maximum spectral resolution (Fig. 3b,c).

Oscillating baroclinic currents are the signature of internal waves. We approximate the baroclinic currents by removing the depth-average current at each time. Internal wave properties in terms of vertical energy propagation direction and rotational polarization are obtained from 2-D fft applied to the time-depth field of baroclinic currents. The result are two-sided frequency ($\omega$) and vertical wavenumber (*k*) spectra, where the polarization is simply given by $\omega$, with $\omega <0$ ($\omega >0$) corresponding to CW (CCW) motion. The ratio of $\omega /k$ is the phase velocity of the wave, with positive values indicating upward phase propagation, and negative values downward^31^. Group velocity and thus energy propagation is 90$^{\circ }$ offset from the phase velocity and has a vertical propagation direction opposite of the phase velocity. The spectra can be categorized into four quadrants according to the permutations of their properties. Inverse fft yields the currents corresponding to the spectral energy in each quadrant.

The criticality parameter ($\alpha$) of a slope with respect to an impinging wave is calculated as:

1 $$\begin{aligned} \alpha (x,y)=\left| \nabla {h(x,y)}\right| \left( \frac{N_{b}^2(x,y)-\omega ^2}{\omega ^2 - f(x,y)^2} \right) ^{1/2} \end{aligned}$$

with the topographic slope $\nabla {h(x,y)}$. In this study, we use the International Bathymetric Chart of the Arctic Ocean (IBCAO) version 4^32^ to obtain topographic slopes. $N_{b}$ refers to the Brunt-Väisälä frequency evaluated at (or close to) the bottom and is obtained from CTD casts performed during the mooring deployment cruise. Although most CTD casts were performed along a single transect along the mooring array (across the continental slope), we use their data for the whole area depicted in Fig. 1b. In the vicinity of a continental slope, properties tend to spread along isobaths, rather than across. We thus use the similarity of bottom depth rather than geographical distance to assign the most representative CTD profile to each grid point.

## Results

### Tidal forcing over the continental slope

Rotary spectra of depth-averaged currents show clear tidal peaks at all moorings (an exemplar is shown for W3 in Fig. 3a). The semidiurnal M$_{2}$ and the diurnal K$_{1}$+P$_{1}$ constituents dominate over other constituents such as O$_{1}$, N$_{2}$ and S$_{2}$+K$_{2}$. Generally, the CW component slightly surpasses the CCW component. DBC are strongest at W2, where maxima appear around November-December and June-July and a minimum around March-April (Fig. 2). This modulation is most likely a consequence of a linear superposition of the two diurnal tidal constituents K$_{1}$ and P$_{1}$: Analogous to the fortnightly spring-neap cycle observed from the interaction of M$_{2}$ and S$_{2}$ tidal constituents, the frequency difference between K$_{1}$ and P$_{1}$ creates en envelope with a period of 183 days. The summer maximum is stronger with current peaks exceeding 15 cm s$^{-1}$ at W2. Current roses indicate, in general, a fairly rectilinear diurnal tidal motion, crossing the isobaths with an angle of $60^{\circ }-70^{\circ }$ (black lines in the current roses show along-slope direction for reference). However, tidal ellipses obtained from harmonic analysis for the K$_{1}$ constituent are much less rectilinear during summer (eccentricity = 0.84) compared to the overall average (eccentricity = 0.92). While the DBC appear barotropic at W2 and W1, at W3 they have a baroclinic component, that might be linked to the topographic trapping of diurnal-period waves at the continental slope.

SBC exhibit fortnightly spring-neap modulation and are strongest at W1 and W2, with no clear longer term variability (Fig. 2). At all moorings, current ellipses associated to SBC are oriented approximately perpendicular to the DBC. At W3, SBC are weaker with the exception of a patch of vigorous currents (>10 cm s$^{-1}$) situated around 500 m to 1000 m water depth between mid-May and mid-August. The energetic patch is subject to fortnightly modulation and coincides in time with the strongest DBC. We use spectral analysis at each depth level to further delineate this signal.

**Figure 4:**
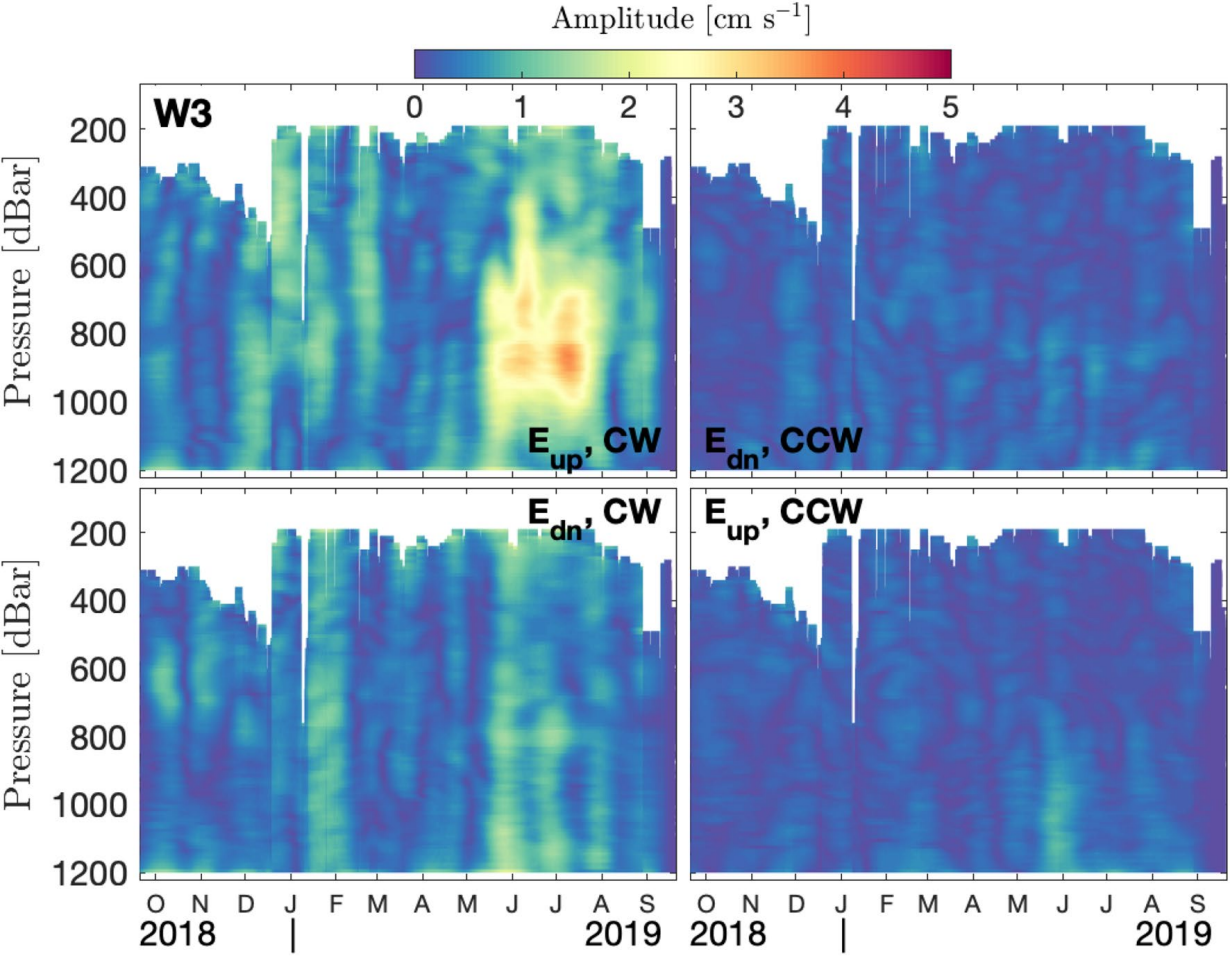
Decomposition of internal wave energy propagation for baroclinic currents in the S$_{2}$+K$_{2}$ frequency band (11.8-12.2h periods) at W3. The four quadrants show different directions (up, down) and polarization (CW, CCW) of internal wave energy propagation. For visualization purposes, we represent the amplitude by plotting the absolute values of these currents.

Spectral analysis over a year-long window permits the differentiation of closely spaced frequencies (such as S$_{2}$ and K$_{2}$) at the cost of losing temporal resolution (and increasing uncertainties of the amplitude). Within the semidiurnal frequency band at W3, there are distinct peaks for major semidiurnal frequencies with sub-inertial M$_{2}$ dominating over the whole water column in both CW and CCW polarization (Fig. 3c). Additionally, there are distinct peaks at super-inertial frequencies S$_{2}$ and K$_{2}$. Surprisingly, these peaks extend over almost mutually exclusive depth ranges: While the S$_{2}$ peak is strongest above 500 m and below 1000 m, the peak at K$_{2}$ frequency is strongest between 500 and 1000 m. This depth range coincides with the strong signal in SBC, observed in summer (Fig. 2). The high spectral power at K$_{2}$ is remarkable, particularly when taking into account the relatively short (several months) duration of the associated SBC signal and the effectively year-long averaging applied in the spectral analysis. We further note that the K$_{2}$ signal is exclusively present for the CW polarized component. Additionally to the CW polarization (Fig. 3), results of 2-D fft analysis reveal that the internal waves associated with the K$_{2}$ summer intensification have amplitudes reaching $\sim$4 cm s$^{-1}$ and propagate upwards (Fig. 4). This effectively rules out generation at the sea surface and suggests a generation mechanism at the seafloor.

### Generation of higher harmonic of K$_{1}$ wave

Direct forcing of the lunisolar semidiurnal constituent K$_{2}$ is exceedingly small (0.45 cm s$^{-1}$ at W3, according to the Arc2km tide model^26^) and thus cannot generate internal waves with an amplitude of $\sim$ 4 cm s$^{-1}$ observed at W3. Instead, our observations suggest that the apparent K$_{2}$ signal is a higher harmonic wave of the strong K$_{1}$ tide (with $2\times \omega _{K_{1}} = \omega _{K_2}$): The topographically trapped diurnal tide (dominated by the K$_{1}$ constituent) reaches cross-shore velocities of $\sim$10 cm s$^{-1}$ during summer at the W2 location near the shelf break (Fig. 2). The interactions between harmonically oscillating currents and topography include the generation of harmonic overtones (i.e. integer multiples of the original frequency $\omega _{K_{1}}$)^15,19,33^. These overtones correspond to high vertical mode numbers and are usually of very small amplitude and subside/dissipate quickly^22^. However, preferential generation of harmonic waves at a specific frequency can be achieved if the aspect ratio of this wave is similar to the aspect ratio of the slope (i.e. the slope is *critical* with respect to that wave)^21^. In the study area, the slope angle is close to critical with respect to $\omega _{K_2}$ over extended stretches (Fig. 1b). Once generated at the continental slope, the super-inertial K$_2$ wave may propagate in offshore (northward) direction. The characteristic slope (*s*) of energy propagation of a freely propagating internal wave emerges from its dispersion relation and is contained in Eq. (1) as

2 $$\begin{aligned} s = \sqrt{(\omega ^2-f^2)/(N^2-\omega ^2)}. \end{aligned}$$

Using this equation and hydrographic data from the CTD profiles, we calculate trajectories of K$_2$ rays originating at the slope (Fig. 1a). The slightly upward propagation trajectory is consistent with the upward energy propagation (Fig. 4). Since the K$_2$ signal ranges between 500 m and 1000 m depth at W3, the origin at the slope has to be somewhat deeper ($\sim$650 m and 1100 m depth). This is not entirely in agreement with the location of critical slope sections on the transect (Fig. 1a). In particular the critical sections at $\sim$500 m do not appear to produce observable signals of K$_2$ at W2 or W3. While the critical sections at $\sim$600 m and $\sim$800 m align well with the K$_2$ signal observed at W3, we would anticipate additional critical points at greater depths than 1000 m at the slope. Although this is not evident on the slope along the mooring section, there is an abundance of critical slopes in the region (as depicted in Fig. 1b). Within 4 km of the mooring array, critical slopes exist at depths corresponding to the probable origin of K$_2$ waves (not shown).

## Discussion

The precise generation site of the K$_2$ wave cannot be determined with the data at hand (effectively only spanning a 2-D section), but there is an abundance of critical slopes in the direct vicinity of the mooring array (Fig. 1b). There must, however, be mechanisms in place that limit the generation and/or propagation of K$_2$ internal waves, as we only observe them within a finite range (between 500 m and 1000 m depths) at a single mooring (W3). The nature of these mechanisms is as of yet unknown.

The variability imposed on the diurnal tidal forcing by the superposition of the diurnal K$_1$ and P$_1$ constituents does not yield two equal peaks: the average cross-slope component of DBC is 6.9 cm s$^{-1}$ in winter, between mid-November and mid-January, and 9.6 cm s$^{-1}$ in summer. The winter peak is thus only about 28% weaker, but does not appear to generate any substantial internal waves in the semidiurnal and K$_2$+S$_2$ frequency bands (Figs. 2, 4). One explanation could be rooted in the quadratic relationship between the amplitude of the second harmonic and the fundamental oscillation that was observed in laboratory experiments^34^: a moderate increase in tidal forcing during summer could lead to a substantially more energetic response in the second harmonic. Additionally, the lack of harmonic generation during winter may also be connected to the pronounced seasonal cycle of the boundary current. In winter, the boundary current reaches its maximum in excess of 40 cm s$^{-1}$ (compared to $\sim$5 cm s$^{-1}$ during summer). The interactions between an along-isobath mean current and oscillations energized at critical frequency are complex and highly non-linear. These interactions are likely to result in distortions, affecting both the amplitude and wavenumber of internal waves^35^. The criticality of the slope and thus generation of higher harmonic waves also depends on stratification (see Eq. 1), but with an average seasonal difference of near-bottom *N*$^2$ of only $\sim 6 \times 10^{-8}$ s$^{-2}$, the effect is negligible in the present case.

The generation of super-inertial internal waves has potential implications for mixing processes and the Arctic internal wave field in general. We show that the internal K$_2$ wave manifests at mid-depth with an amplitude of 4 cm s$^{-1}$ (Fig. 4), that combines with the ambient semidiurnal tide to in excess of 10 cm s$^{-1}$ in a fortnightly modulation (Fig. 2). Such sub-surface shear may be substantial enough to overcome the weak Arctic Ocean interior stratification and generate mixing and associated vertical heat fluxes from the AW layer. In the vicinity of the shelf break, the K$_2$ wave may play a role in the lateral export of particles (within a turbid layer) and carbon from the shelf seas such as observed for the Barents Sea^36^ and Laptev Sea^37^. Importantly, these impacts may not be constrained to regions immediately adjacent to the continental slope if the super-inertial waves radiate freely away. This is supported by 2-D theory, where waves propagate freely as soon as their frequency becomes super-inertial^21^. However, 3-D model results indicate that the transition from trapped along-slope propagation to free propagation is gradual, with weakly super-inertial waves (such as K$_2$, with $\omega _{K_2}=1.01\times f$) still preferentially propagating along topography with only a minor offshore component^14^. The extent to which the waves we observe are relieved from the topographic waveguide is thus unclear.

If their longevity and freely propagating energy is substantial, these waves would become part to the background internal wave field in the Arctic Ocean, and contribute to Arctic-wide interior background mixing by the cascade of energy from internal waves down to molecular mixing.

Observations from Arctic shelf seas show that sea-ice cover acts as a damper for tidal elevation and tidal currents^38,39^. With the Arctic Ocean sea-ice cover rapidly declining, tidal currents may be expected to increase, potentially boosting processes dependent on vigorous tidal current forcing like the generation of higher harmonics at the continental slopes described here. A consequence could be more energetic super-inertial internal waves traversing the Arctic Ocean and/or a geographically more widespread generation of these waves.

## Summary

Year-long velocity observations from three moorings deployed across the continental slope north of Svalbard show evidence for super-inertial internal waves at K$_2$ frequency. Frequency, timing, polarization, propagation direction of these waves are consistent with the generation as higher harmonics of the topographically trapped, sub-inertial K$_1$ tide at critical slope angles. The amplitude of these waves reaches 4 cm s$^{-1}$, but combines in linear superposition with the ambient semidiurnal tide to in excess of 10 cm s$^{-1}$ in a fortnightly modulation. The shears associated with this signal can be substantial with implications for local mixing. While the weakly super-inertial waves would likely preferentially propagate along topography, they have the potential to propagate offshore and might contribute to mixing processes far away from the slope. However, the extent of the longevity of these waves is unknown.

## Data availability

The Nansen Legacy mooring data (W1-W3) are available from the Norwegian Marine Data Centre: https://doi.org/10.21335/NMDC-1852831792^28^. The CTD data from the mooring service cruise 2018 (KH2018709) is also available from the Norwegian Marine Data Centre https://doi.org/10.21335/NMDC-2039932526^30^. The Arctic 2 km Tide Model (Arc2kmTM) can be downloaded from the Arctic Data Center at https://doi.org/10.18739/A2PV6B79W^26^. Topographic data from the International Bathymetric Chart of the Arctic Ocean Version 4 is available under https://www.gebco.net/about_us/committees_and_groups/scrum/ibcao/ibcao_v4.html^32^. The model calculating mode shapes for trapped waves can be accessed at https://github.com/hugke729/RidgeTrappedWave^25^.
